# Personality and achievement along medical training: Evidence from a cross-lagged analysis

**DOI:** 10.1371/journal.pone.0185860

**Published:** 2017-10-17

**Authors:** Isabel Lourinho, Maria Amélia Ferreira, Milton Severo

**Affiliations:** Department of Medical Education and Simulation, University of Porto, Faculty of Medicine, Porto, Portugal; Universidade de Mogi das Cruzes, BRAZIL

## Abstract

**Introduction:**

Evidence on personality traits change implies it should be studied as an outcome and not only as an explanatory effect. Therefore, we aimed to assess how personality and academic achievement sway each other. Three cohorts of medical students (n = 181) comprised of school leavers and graduates, completed NEO-FFI when admitted (baseline) and later on medical training (follow-up). Previous achievement was measured as mean scores on national school examinations, and academic achievement as medical course average. Causal relations were studied by cross-lagged analysis.

**Results:**

Cross-sectional analysis at baseline showed differences between graduates and school leavers on personality, with graduates scoring lower on neuroticism (β = -12.344, p<0.001), and higher on openness to experience (β = 5.257, p<0.001), conscientiousness (β = 2.345, p = 0.004,) and agreeableness (β = 6.993, p<0.001). Longitudinal analyses indicated that personality traits and achievement tracked over time. Cross-lagged analysis found a positive significant association between academic achievement and neuroticism at baseline (β = 0.031, p = 0.014) and with being a graduate student (β = 0.766, p = 0.006). After adjusting, no association was found between previous achievement and personality at follow-up.

**Conclusions:**

Some neuroticism may enhance medical academic achievement. The blurring of the initial differences between graduates and school leavers suggests a reasonable possibility of personality traits change along the medical course.

Future research on medical selection processes cannot afford to ignore the influence of the medical school environment on personality traits change.

## Introduction

The selection of medical students is under debate for years and yet it seems to be an unresolved challenge [1]. Tomorrow’s doctors are required to express specific skills and personal attributes besides being well qualified and competent technicians [2]. This paradigm shift in academic medicine has had effects in the selection processes and in some medical schools’ curriculums [3]. For many years, selection has relied heavily on previous achievement, shown to be important for progression through medical school and beyond, so that applicants admitted on the basis of their previous achievement had lower levels of dropout[4–6]. However, it cannot be assumed that those with high academic ability alone can be turned into competent physicians via medical training, as personal characteristics may need to be present from the beginning [7]. In recent years, there has been a proliferation of medical selection tools that aim to pursuit non-academic characteristics among the academically well-qualified applicants, such as interviews [8], mini-multiple-interviews (MMI) [9] or situational judgement tests[10]. Also, it has been shown how different medical selection tools can call upon different personality traits [8,11].

The influence of personality traits on important life outcomes such as health, well-being, job performance and career success [12,13] has contributed to the proliferation of personality assessment in different fields of knowledge. In the medical education field, the five-factor model (FFM), or Big Five, is one of the most used [14]. FFM comprises the broad trait dimensions referred to as OCEAN: openness to experience, conscientiousness, extraversion, agreeableness, neuroticism (or emotional stability which is the opposite of neuroticism) and their more specific facets [15]. For example, personality traits have already been linked to empathy in medical students [16] and to mental health [17]. As regarding the association of personality traits with medical academic achievement, the personality trait of conscientiousness appears to be a predictor of success [18]. Yet this association is not linear because there is some evidence that this trait may be positively associated with knowledge-based assessment, but negatively associated with some clinical aspects of medical school assessment [7]. There are also some concerns that personality tests are ‘fakeable’ when used for medical student’s selection. Indeed, past research had already shown that individuals can fake in line with the requirements of particular jobs[19,20]. Faking consists of the deliberate false presentation of the self that may be favourable (fake good) or unfavourable (fake bad) [21]. In addition, a recent study has suggested that graduate applicants who have participated in a medical school selection process, have faked on self-report personality tests [22].Account must be taken of the fact that some of these studies, besides having a cross-sectional design, have conceptualized personality traits only as explanatory variables. This conceptualization is in accordance with a perspective of personality traits as deterministic and stable all over a lifetime and that do not develop through experience [23]. However, there has been evidence that personality traits show meaningful and statistically significant mean-level change in young adulthood, middle age and even at old age [24]. Built into this principle is the assumption that personality traits remain open systems that may be influenced by the environment. This does not mean that they are necessarily influenced by the environment or that they must change, but rather that they have the capacity for change at any age [23]. Personality traits change seems to be more predominant in young adulthood [25], and specific positive or negative life experiences, life goals, abilities and social roles may be some of the presumed causes of changing [26,27]. Thus, it is required that any predictive model must integrate the assumption that personality traits remain changeable throughout the life cycle.

Recently, it has been identified the lack of data on personality change and its implications for healthcare professional’s health, performance but also for the medical selection research. The selection of a specific trait is itself of limited validity in the face of dynamic trait change and context specificity of trait expression [28]. Hence, for this study, we adopted a position in agreement with personality development theories wherein the person variables and social situations are both integrated [23,24]. In methodological terms, it implies that personality traits must also be studied as outcomes and not only as explanatory effects. For this purpose, we used a cross-lagged panel design because it allows at least two assessments at different time points. Therefore, we aimed to assess how personality and medical academic achievement sway each other.

## Material and methods

### Contextualization

Admission to a Portuguese medical school is extremely challenging and competitive because, as it happens in other countries, demand exceeds supply. From a historic perspective, admission to medicine has been dominated by young school leavers, typically aged 18–19 years. School leaver’s selection is a national seriation process that relies exclusively on previous achievement. This quantitative variable consists in student’s secondary classifications and exit national examinations (Mathematics, Chemistry and Biology). Applicants are ranked according to their previous achievement and apply for the existent medical schools.

A decree law established in 2007 that every medical school had to have a graduate entry mode apart from the school leaver’s selection process. From the existent seven public medical schools, only one is exclusively for graduate students. The other medical schools have both school leavers and graduate quotas, and are autonomous to decide on the selection practices to adopt in the graduate entry approach. For example, while some medical schools have adopted a written examination followed by a MMI, others have chosen the combination of previous achievement and admission interview.

For the last decades, the Faculty of Medicine of the University of Porto (FMUP), has been the first option of the school leavers in Portugal presenting the highest access ratings. This medical school is 192 years old and has had a traditional medical curriculum until the academic year 2013/2014, when a curricular reform was undertaken. This six-year medical course credits entitles the graduation in Basic Health Sciences after the completion of the first 180 ECTS credits. The conclusion of 360 ECTS credits for 12 semesters confers the MSc in Medicine.

FMUP has 245 places available per year for school leavers and 37 for the graduate students. At the time of this study, the graduate entry selection practices comprised a two-phase process: previous achievement and admission interview. The first phase ranked applicants based on their previous achievement. The highest achievers were eligible for the admission interview that aimed to pursue personal characteristics that go beyond academic success [8].

Although some medical schools adopted a specific curriculum for graduates, this did not occur at FMUP. At our faculty, all students attend the same medical course, even though graduates may have some recognition of previous academic performance.

### Flow of the participants

**Fig 1:**
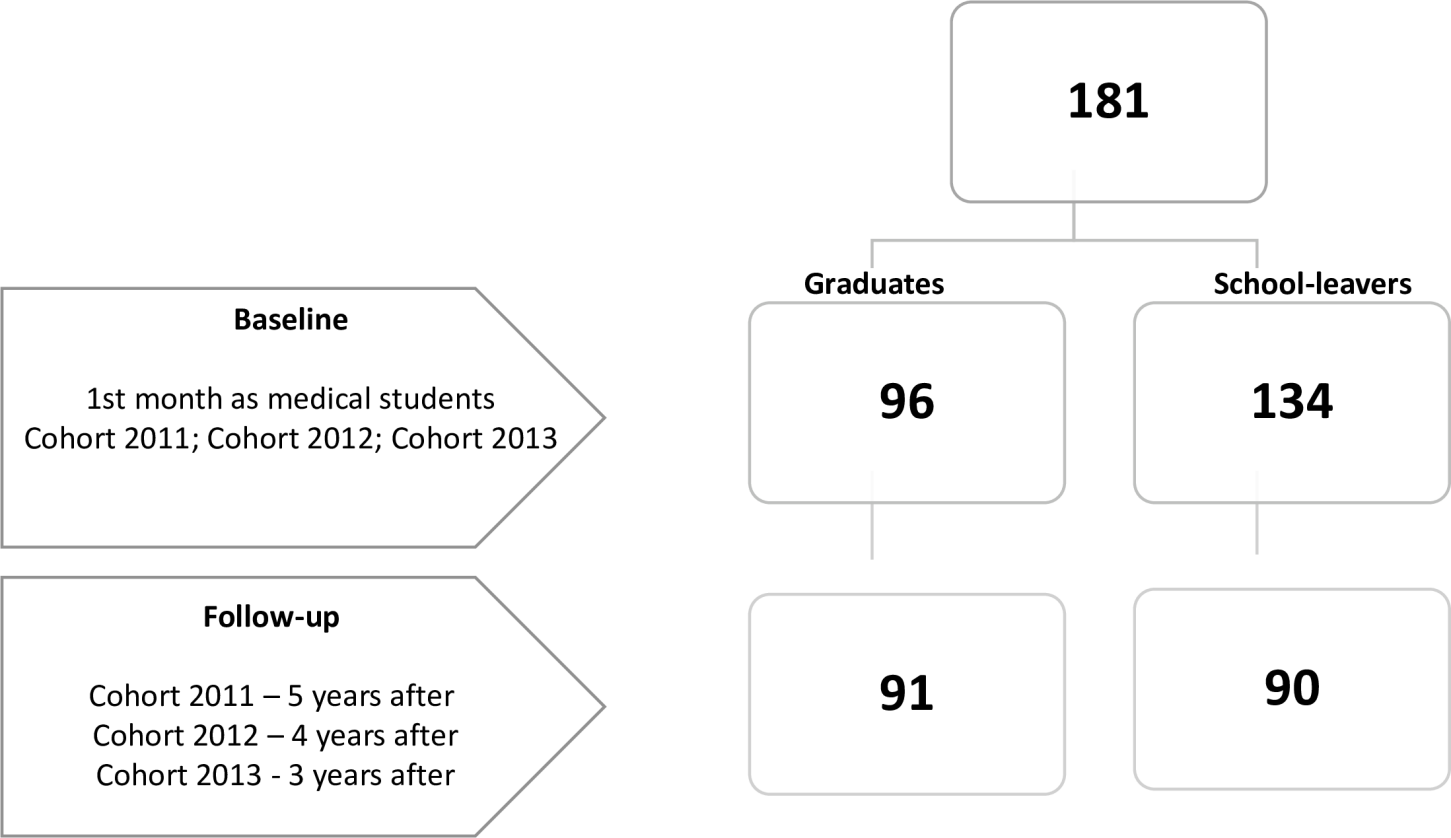
Flow of the participants.

We used information from three cohorts of graduate students and for each graduate we selected one school leaver admitted on the same academic year. Baseline assessment occurred during participant’s first month as medical students at FMUP. They were asked to complete a personality measure (as part of a larger battery of psychometric tests). Follow-up assessment occurred in April 2016, which means that the first cohort was assessed 5 years after, the second cohort was assessed 4 years after and the third cohort was assessed 3 years after having been admitted to medicine. Participants voluntarily completed the same psychometric tests again (Fig 1).

At baseline and at follow-up assessments, questionnaires were sent by e-mail and participants had two weeks to answer in their own time, unsupervised. Participants were asked to respond honestly on both assessments and no manipulated instructions were given.

### Participants

From the 3 cohorts of medical students comprised of secondary school leavers and graduates who were admitted to FMUP between 2011 and 2013, a total of 181 medical students participated in this study (Fig 1). At baseline assessment, from the 111 eligible graduates, 96 (86. 5%) participated, and from the 146 school leavers we invited to collaborate, 134 (91.8%) agreed to participate in the study. At follow-up, from the 96 graduate participants at baseline, 91 collaborated once again, and from the 134 school leavers 90 (67.2%) also participated (Fig 1). From 181 participants, 49.7% were graduate and the majority were women (65.7%).

### Measures

Personality traits were assessed through the short version of the NEO Personality Inventory (NEO-PI-R), which is called the NEO Five-Factor Inventory (NEO-FFI). This 60-item, multiple-choice questionnaire evaluates five main dimensions of personality: openness to experience, conscientiousness, extraversion, agreeableness and neuroticism in a five-point Likert scale that ranges from 0 (strongly disagree) to 4 (strongly agree). Additionally, the NEO-FFI had already been validated for the Portuguese population [29].

### Previous achievement

Previous achievement was measured as the mean scores of every participant exit national school examinations on Mathematics, Chemistry and Biology. Scores were between 0 and 200. The Z-scores transformation (subtract the mean of the examinations and divided by the respective standard deviation) was applied to each score and the mean of the 3 examinations was then calculated.

### Academic achievement

Academic achievement was measured as the medical course average. If the first three years are mainly Basic Sciences theory oriented, thereafter there is a high clinical focus. From the fourth year onward, medical students attend clinical practice units that require close contact with patients. These clinical practices take place at FMUP affiliated health facilities, mostly based in the north of Portugal. They include public hospitals (S. João Hospital centre is the most interactive one), private hospitals and health centres. Subjects in which graduates have had previous recognition due to their past academic or professional experience were discarded for this variable design, and the mean of the scores was calculated for each graduate.

### Data analysis

**Fig 2:**
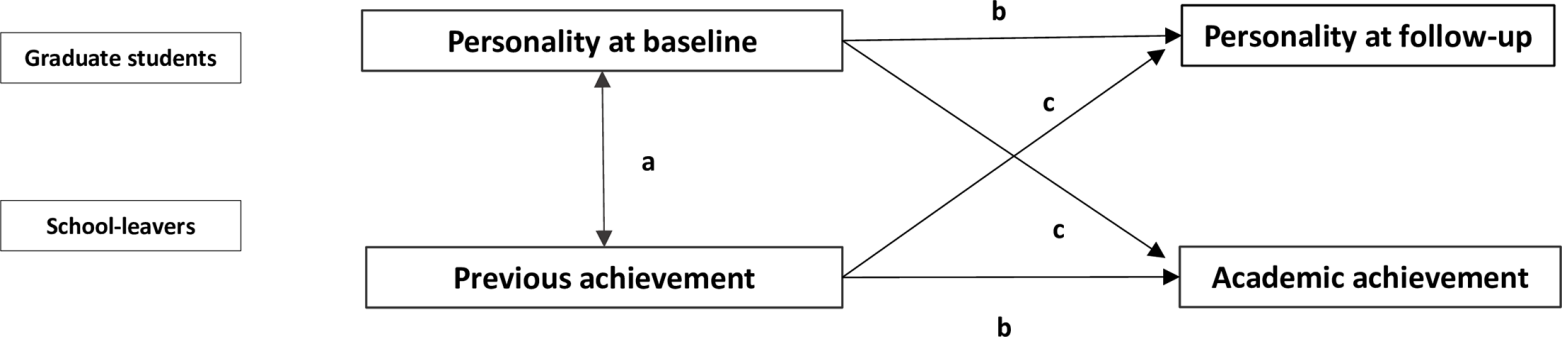
Conceptual model of the cross-lagged association between personality and achievement.

To estimate the association between the exposure and outcomes, linear regression models were used to calculate regression coefficients (β) and the respective 95% confidence intervals (95% CI). Interaction terms were tested between achievement and personality traits, but also between graduates and the previous variables. Additionally, a cross-lagged panel design analysis was performed to investigate the causal relations [30]. The conceptual model is described in Fig 2. This model includes three linear regressions: a) a cross-sectional analysis between personality traits at baseline and previous achievement; b) a longitudinal analysis in which we performed a linear regression between personality traits at follow-up and personality traits at baseline, and between academic achievement at follow-up and previous achievement at baseline; c) a cross-lagged analysis between personality traits at baseline and academic achievement at follow-up.

For linear regression models, homoscedasticity and normality of errors distribution were evaluated. In all analyses, we considered a p value <0.05 as statistically significant. Statistical analysis was conducted using SPSS statistical software package version 21 (SPSS Inc., Chicago, IL, USA).

### Ethical considerations

This study was approved by the Ethics Committee of the Faculty of Medicine of the University of Porto and Hospital de São João, and policies and procedures were developed to guarantee data confidentiality and protection. Participants received written and oral information explaining the purpose and the design of the study and written informed consent was obtained.

## Results

We found a positive association between previous achievement and the neuroticism trait at baseline (β = 4.578, p<0.001, Table 1). With regard to previous achievement and the other personality traits at baseline, negative associations were found with openness to experience, conscientiousness and agreeableness traits (β = -1.533, p = 0.004; β = -1.398, p = 0.008; β = -2.590, p<0.001, respectively). No associations were found between previous achievement and the extraversion trait (β = -0.605, p = 0.220, Table 1).

**Table 1:** Cross-sectional association between personality traits and previous achievement at baseline.

		Crude β (95CI %)	P
Personality traits at baseline			
Openness to experience			
	Previous achievement	**-1.533 (-2.573, -0.494)**	**0.004**
	Graduates	**5.257 (3.808, 6.706)**	**<0.001**
Conscientiousness			
	Previous achievement	**-1.398 (-2.430, -0.366)**	**0.008**
	Graduates	**2.345 (0.755, 3.936)**	**0.004**
Extraversion			
	Previous achievement	-0.605 (-1.576, 0.365)	0.220
	Graduates	-0.315 (-1.837, 1.208)	0.684
Agreeableness			
	Previous achievement	**-2.599 (-3.660, -1.535)**	**<0.001**
	Graduates	**6.993 (5.570, 8.415)**	**<0.001**
Neuroticism			
	Previous achievement	**4.578 (2.760, 6.396)**	**<0.001**
	Graduates	**-12.344 (-14.719, -9.970)**	**<0.001**

Graduates showed lower levels in the neuroticism trait at baseline when compared to school leavers (β = -12.344, p<0.001, Table 1), but scored higher in the personality traits of openness to experience (β = 5.257, p<0.001, Table 1), conscientiousness (β = 2.345, p = 0.004, Table 1) and agreeableness (β = 6.993, p<0.001, Table 1). There was an interaction between graduates and previous achievement on personality traits (data not shown).

In the longitudinal approach, it was observed that personality traits and achievement tracked over time. Every personality trait assessed at baseline was significantly positively associated with the same trait assessed at follow-up: openness to experience (β = 0.273, p<0.001, Table 2), conscientiousness (β = 0.225, p<0.001, Table 2), extraversion (β = 0.174, p = 0.012, Table 2) agreeableness (β = 0.315, p<0.001, Table 2) and neuroticism (β = 0.406, p<0.001, Table 2). Previous achievement was significantly positively associated with posterior academic achievement (β = 0.352, p = 0.007, Table 3).

**Table 2:** Cross-lagged associations between personality traits and previous achievement at follow-up.

Outcome	Exposure	Crude β (95CI %)	P	Adjusted β (95CI %)*	P
Personality traits at follow-up					
Openness to experience					
	Openness to experience at baseline	0.225 (0.115, 0.336)	<0.001	**0.273 (0.143, 0.402)**	**<0.001**
	Previous achievement	-0.519 (-1.339, 0.300)	0.213	-0.559 (-1.486, 0.368)	0.236
	Graduates	0.597 (-0.659,1.843)	0.349	-1.132 (-3.07, 0.812)	0.252
Conscientiousness					
	Conscientiousness at baseline	0.267 (0.150, 0.383)	<0.001	**0.0225 (0.092, 0.359)**	**0.001**
	Previous achievement	-0.589 (-1.458, 0.278)	0.181	0.259 (-0.699, 1.217)	0.485
	Graduates	1.779 (0.445, 3.113	0.009	1.658 (-0.352, 3.669)	0.105
Extraversion					
	Extraversion at baseline	0.215 (0.098, 0.332)	<0.001	**0.174 (-0.086, 0.211)**	**0.012**
	Previous achievement	-0.083 (-1.675, -0.091)	0.029	-0.226 (-0.699,1.217)	0.608
	Graduates	1.630 (0.425, 2.834)	0.008	0.871 (-0.353, 3.669)	0.346
Agreeableness					
	Agreeableness at baseline	0.330 (0.231, 0.428)	<0.001	**0.315 (0.166, 0.463)**	**0.001**
	Previous achievement	-0.895 (-1.727, -0.064)	0.004	-0.026 (-0.911, 0.859)	0.954
	Graduates	0.234 (0.983, 3.485)	0.001	0.267 (-1.597, 2.131)	0.778
Neuroticism					
	Neuroticism at baseline	0.372 (0.285, 0.460)	<0.001	**0.406 (0.279, 0.534)**	**<0.001**
	Previous achievement	1.300 (-0.044, 2.645)	0.058	0.046 (-1.279, 1.371)	0.945
	Graduates	-2.714 (-4.762, -0.665)	0.009	**2.627 (-0.163, 5.418)**	**0.065**

*Adjusted for all personality traits at baseline, previous achievement and graduates.

**Table 3:** Cross-lagged associations between academic achievement and personality traits at baseline.

Outcome	Exposure	Crude β (95CI %)	P	Adjusted β (95CI %)*	P
Academic achievement at follow-up					
	Openness to experience at baseline	0.000 (-0.031, 0.033)	0.955	-0.010 (-0.046; 0.026)	0.581
	Conscientiousness at baseline	0.014 (-0.018, 0.046)	0.399	0.018 (-0.018;0.054)	0.311
	Extraversion at baseline	-0.008 (-0.431, 0.026)	0.63	0.006 (-0.034; 0.046)	0.777
	Agreeableness at baseline	0.0144 (-0.016, 0.044)	0.344	0.019 (-0.024; 0.063)	0.385
	Neuroticism at baseline	0.007 (-0.010, 0.025)	0.394	**0.031 (0.006; 0.561)**	**0.014**
	Previous achievement	0.189 (-0.036, 0.415)	0.099	**0.352 (0.095; 0.610)**	**0.007**
	Graduates	0.241-(0.11, 0.591)	0.177	**0.766 (0.223; 1.309)**	**0.006**

*Adjusted for all variables.

At follow-up a positive association between neuroticism and being a graduate student was found (β = 2.627, p = 0.065, Table 2).

The crude association at follow-up showed that, when compared to school leavers, graduates scored higher on the personality traits of agreeableness and extraversion (β = 0.234, p = 0.001; β = 1.630, p = 0.008, Table 2) and scored lower on the neuroticism trait (β = -2.714, p = 0.009, Table 2). After adjusting, the difference between both populations was smaller than at baseline (β = -12.344, p<0.001) with graduate students scoring a little more on the neuroticism trait than school leavers (β = 2.627, p = 0.065, Table 2).

Cross-lagged analysis showed a positive significant association between being a graduate student and academic achievement (β = 0.766, p = 0.006, Table 3). In the cross-lagged association between personality traits assessed at baseline and academic achievement at follow-up, a positive significant association was found between the neuroticism trait at baseline and academic achievement (β = 0.031, p = 0.014, Table 3). No other significant associations were found for the other personality traits (Table 3). Regarding the cross-lagged association between previous achievement and personality traits at follow-up, it was not significant for any personality trait (Table 2).

## Discussion

On the basis of our conceptual model (Fig 2), this study has confirmed the cross-lagged association between personality at baseline and academic achievement at follow-up. However, it did not confirm the cross-lagged association between previous achievement and personality at follow-up. It was also found that personality traits and achievement tracked over time. In addition, graduates and school leavers showed different personality traits at the beginning, but along the medical course it seems that graduates have increased their neuroticism and academic achievement.

Heterogeneity of personality traits between school leavers and graduates population at baseline has been shown with the latter, scoring higher on openness to experience, conscientiousness and agreeableness and lower on the neuroticism trait. This result corroborates studies that found that graduates may contribute to widen psychological diversity [31]. Still, it must be mentioned that these graduates are the same who had scored higher on the traits of openness to experience, extraversion and conscientiousness in their admission interview [8]. This fact reinforces that different medical selection tools call upon different personality traits among applicants [11]. Although this heterogeneity seems to keep the same direction at crude cross-lagged analysis, after adjusting, graduates have increased their neuroticism scores. It must be mentioned that despite the mean levels of neuroticism have increased at follow-up it were still lower than the other personality traits.

The association between personality traits with previous achievement at baseline, irrespective of whether one was a graduate or a school leaver, confirms that previous achievement can be a surrogate variable of personality traits. In addition, previous achievement was negatively associated with openness to experience, conscientiousness and agreeableness, and positively associated with neuroticism, with the association being maintained at the crude cross-lagged analysis. However, after adjusting for the tracking effect (longitudinal analysis has shown that all outcomes—personality and achievement- tracked over time), previous achievement no longer had any effect on personality change, meaning that it is not a personality change explanatory fact.

The cross-lagged analysis has shown that medical academic achievement can be determined by the neuroticism trait at baseline, being a graduate student, and previous achievement. On one hand, this study confirms the extensive research about the positive association between previous achievement and academic achievement [32–34]. On the other hand, unlike previous studies, we have not found the conscientiousness trait to be a predictor of medical academic achievement [35,36], but rather the neuroticism trait. This is an important result as it has been mooted that conscientiousness is considered a key trait when selecting medical students [33]. Yet this is not an absolute novelty. Tett had already shown that being conscientious is not always beneficial because detail-oriented people may take a very long time compromising fast decision-making [37]. More recently, it has been evidenced that the association between one conscientiousness and learning outcomes may change in direction (from enhancing to inhibiting) as context changes. All together led to a growing awareness that traits like conscientiousness also have a ‘dark side’ and traits like neuroticism, have a ‘bright side’ [38]. Or according to the evolutionary theory of personality, every trait has simultaneously its ‘costs and benefits’. The neuroticism personality axis is associated with variation in the activity levels of negative emotion systems such as fear, sadness, anxiety, and guilt. However, if the negative effects of neuroticism are well-known in the psychological literature its benefits are not so widespread [2]. The fact is that neuroticism seems to be positively correlated with competiveness [3] and among university students, it has been shown to be positively associated with academic achievement [4, 5]. A longitudinal study in a Swedish upper secondary school sample has found the same association [39]. In addition, in the medical education research it has also been highlighted that those who have moderately higher levels of neuroticism perform better on anxiety-provoking part of the course [38]. It is possible that the anxiety component of neuroticism due to its anticipatory ability can have facilitated performance in some of these individuals who may have strategically channelled the negative affect to promote high levels of preparation, competitiveness, and striving to attain a better position [37][38][34][33]. This behaviour reflects greater vigilance and supports the tendency to move towards the object of anxiety to control it[40]. This means that it seems better to be a bit neurotic than to be stable in what regards getting good marks.

Notwithstanding these benefits of some levels of neuroticism that can serve as a motivator in the competitive medical school environment, we cannot forget its evident drawbacks. Its negative effect as a strong predictor of stress and burnout among medical students and doctors is well-known [17,41,42]. In addition, a prevalence of poorer mental health has been shown among medical students when compared with other populations of the same age [43]. It is also important to highlight that a successful medical student won’t be necessarily a competent or a healthy/happy doctor [7,44]. Moreover, these medical students were assessed either in the initial years of the medical course or in their clinical years with more patient care experience. This has made us reflect if the available assessment methods on the medical course do privilege cognitive performance. Another interesting fact of this study was the positive relationship between being a graduate student and medical academic achievement. We know that most of these graduates were not admitted to medicine while school leavers were admitted because of their previous achievement (data not shown). According to a neo-socioanalytic theory, the social roles are the primary conduit through which environment affects personality. The ability to model (watching others) is also embedded in roles such as those found at medical school. This can be seen in the formal mechanism of performance feedback. It is possible that graduate students may be under the impression that they are performing well (identity perception), until their professors/ school leaver colleagues provide less than flattering performance feedback (reputation). Similar feedback mechanisms may affect change in traits and interests as people acquire information about their social reputation from others in the environment. Hence, changes can occur through watching ourselves do things differently, often in the context of a new role or in response to new role demands [23]. It is also possible that, due to their previous experiences, graduates have further valued their role as medical students [45] and have engaged in task-oriented behaviours to avoid the threat of appearing incompetent to others [46]. In some way, the fear of failure or of causing a bad impression can enhance medical academic achievement. Furthermore, graduates were the population in which neuroticism levels increased the most when compared to school leavers. Taken together, these findings suggest that the medical school environment seems to blur the boundaries between two totally different populations at baseline. We agree that the intensity and nature of medical training is very likely to result in personality change [27] and that it is also possible that the medical school context can elicit the ‘dark-side’ and ‘bright-side’ aspects of personality traits [28].

Some critical issues should be taken into consideration when interpreting the results of this study. First, we must acknowledge that it is a single-centre study, which could make it unclear as to whether these findings could be generalised to other settings. However, FMUP has had the highest access ratings for secondary school leavers in Portugal over the last decades, being the first medical school option for the majority of secondary school leavers. Furthermore, at least 12% of our graduate applicants are common to other three Portuguese medical schools that have different selection criteria for the graduate entry mode, whereby there is confidence that, at the very least, these findings can be extrapolated to Portugal. Another limitation is related with our participants’ age. Although we have a sample comprised of graduates and school leavers, personality traits change seems to be more robust in young adulthood (age 20–40)[24]. This means that it is not possible to separate what pertains only to a person’s stage of life or to the medical school environment. The third limitation is concerned with assessments. The national examinations which form the previous achievement variable may have some variance on its difficulty per year. But more importantly, previous achievement was not assessed at the same time as personality traits. Whereas for most school leavers it was only a two-month time difference, for graduates, as previous achievement was built with national secondary examinations, it may have occurred years before. These years may have contributed to a difference in personality scores. Nevertheless, given that this was the selection criteria at the time, there was no possibility to circumvent this limitation. Moreover, we opted to present the data of the three cohorts measured as only one cohort because we had not enough sample power to study the effect stratified by cohort. Nevertheless, we must say that we did perform the sensitivity analysis and despite the lost sample power, we did confirm that results had the same trend. Finally, it may also have occurred that individuals faked self-report personality tests but as they completed the NEO-FFI after being admitted to the medical school, we assume participants answered honestly.

This study’s major strength is its cross-lagged analysis that enables to investigate the causal relations between variables. Moreover, this study design allowed the conceptualization of personality traits also as outcomes in accordance with a dynamic perspective between the person and the situation. Unlike previous research, the personality variable was assessed twice and not only once. Also, our sample was comprised of two selected populations, which enables to study the influence of the medical environment on personality traits change. In addition, these cohorts continue to be followed and other psychological dimensions are being assessed.

In conclusion, this study confirms previous research on the association between previous achievement and medical academic achievement, the selection of different personality traits through different medical selection tools, and that graduates can widen psychological diversity among medical students. Nevertheless, it adds that some neuroticism may enhance medical academic achievement, reinforcing that all personality traits have simultaneously its bright and its dark-sides. Finally, the blurring of the initial differences between graduates and school leavers, suggests a reasonable possibility of personality traits change along the medical course. To support this finding, it would be valuable for this study to be replicated elsewhere. Medical students’ selection process remains an unresolved challenge, but future research cannot afford to ignore the influence of the medical school environment on personality traits change.

## Supporting information

S1 Dataset (CSV)
